# Corrigendum: Prevalence and Associated Factors of Internet Addiction Among Undergraduate Students at Al-Beheira Governorate, Egypt

**DOI:** 10.3389/ijph.2022.1605567

**Published:** 2022-12-09

**Authors:** Basem Salama

**Affiliations:** ^1^ Department of Community Medicine, Damietta Faculty of Medicine, Al-Azhar University, Cairo, Egypt; ^2^ Faculty of Medicine, Northern Border University, Arar, Saudi Arabia

**Keywords:** internet, addiction, undergraduate, students, behavior, adolescent, prevalence, risk factors

The authors requested correction of two errors in the affiliations of Basem Salama in the article published 2020.

1) Instead of “Community Medicine Department, Damietta Faculty of Medicine, Al-Azhar University, New Damietta, Damietta Governorate, 34511, Egypt”, the correct affiliation was “Department of Community Medicine, Damietta Faculty of Medicine, Al-Azhar University, Cairo, Egypt”.

2) In addition to affiliation 1, Baseem Salama was also affiliated with “Faculty of Medicine, Northern Border University, Arar, Saudi Arabia.”

The author apologizes for these errors and state that this does not change the scientific conclusions of the article in any way.

